# T Cell Subpopulations in Healthy Elderly and Lung Cancer Patients: Insights from Cuban Studies

**DOI:** 10.3389/fimmu.2017.00146

**Published:** 2017-02-14

**Authors:** Danay Saavedra, Beatriz Garcia, Agustin Lage

**Affiliations:** ^1^Clinical Immunology Department, Center of Molecular Immunology, Havana, Cuba

**Keywords:** CD8 T cells, late-stage differentiated CD8 T cells, non-small cell lung cancer, cancer vaccine, CMV

## Abstract

The senescence of the immune system and the risk of cancer increase with aging. Age itself entails changes in the immune system, which are related to a decrease in thymic output of naïve lymphocytes, an accumulation of chronic antigenic load, notably chronic viral infections such as cytomegalovirus (CMV), and replicative senescence of lymphocytes. These changes could eventually contribute to cancer risk and affect the response to cancer treatment. However, several confounding factors make it difficult to draw a picture of causal relationships. Studies in diverse human populations could contribute to clarify these complex relationships. Here, we summarize the current knowledge about the senescence of the T cells, the relationship with CMV infection, cancer, and cancer treatment. We also review the results of a series of studies performed in Cuba whose population is characterized by the unusual combination of long life expectancy and high antigenic load, including high seroprevalence of CMV, typical of tropical countries. Although immunosenescence affects almost all components and functions of the immune response, its most salient feature is a decrease in numbers and proportions of naïve CD8^+^ T lymphocytes and an accretion of terminally differentiated CD8^+^ T lymphocytes. These features were confirmed by the Cuban studies, but interestingly a clear gender effect also appeared. Moreover, as aging is a global phenomenon, a fast increase in elderly with malignancies is expected; therefore, the evaluation of patient’s immune status would support the decision of treating them with immunotherapy and predict the efficacy of such treatments, thereby improving benefits for the patients.

## Introduction

Aging is related to changes in innate and adaptive immune system. Those age-related changes could be associated with susceptibility to infectious diseases, Alzheimer’s disease, autoimmunity, osteoporosis, and cancer (1).

However, several potentially confounding factors make it difficult to draw a clear picture of causal relationships. For example, the risk of cancer increases with age and cancer itself. Cancer treatment could also influence the immune system and chronic infections such as cytomegalovirus (CMV) could drive immunosenescence. These processes occur simultaneously; nevertheless, it is not yet determined in what magnitude they are causally related (2).

The changes most consistently found in immunosenescence studies pertain to CD8^+^ T cells. It is well documented that with decrease in age naïve CD8^+^ T cells, highly differentiated CD8^+^ T cells lacking CD28 accumulate and those CD8^+^CD28^−^ T cells upregulate the expression of CD45RA. Additionally, these cells show signs of replicative senescence such as a decreased proliferation ability, shortened telomeres, impairment of telomerase activity, and upregulation of CD57 (3, 4). These facts have been frequently associated with chronic CMV infection (5).

The relationship between these changes and a susceptibility to disease was first documented in the Swedish octogenarian study (OCTO-immune). This study defined the concept of “immune risk profile” (IRP). The IRP was described as a decrease in number and frequency of B cells, an increased count of CD8^+^ memory T cells, a CD4/CD8 ratio of less than 1, and a rise of CD8^+^CD28^−^ among other late-stage differentiated T cells (6). Additionally, most of these late-stage differentiated cells express CD57 (7). The IRP was also associated with high serum concentrations of pro-inflammatory cytokines and seropositivity to CMV (6, 8).

High prevalence of CMV is found worldwide; however, it fluctuates depending on the region (9). The CMV infection frequently starts during adolescence and persists throughout life. The expansion of CD8^+^CD28^−^ T cells upregulating CD57 has been associated with CMV infection. It is considered one of the main causes of immunosenescence (8).

A possible connection between the high prevalence of CMV infection in tropical countries and some sociocultural behaviors, which contribute to CMV transmission in early stages of life, has been postulated (10). Our group confirmed a high seroprevalence of CMV infection in healthy Cubans from an early age (4).

In the present paper, the current knowledge on the dynamics of T cell subpopulations during aging is reviewed, as well as the relationship with CMV infection, cancer, and cancer treatment. The results of several studies carried out in Cuba are also interpreted. As Cuban population presents an unusual combination of a long life expectancy and high antigenic load of a tropical country, it is a kind of natural experiment, which could show novel aspects in the relationships between immunosenescence and chronic non-communicable diseases.

## The Increased Proportion of Late-Differentiated CD8 T Cells in Healthy Cubans is Influenced by Age and Gender

Long-lasting antigenic stimulation causes the progressive increase of late-stage differentiated, oligoclonal T cells, mainly, but not exclusively, within the CD8^+^ T cell compartment. Increasing evidence demonstrate that the CD8^+^CD28^−^ and CD8^+^CD57^+^ T cell populations play an essential role in innumerable diseases or chronic inflammation-related conditions, associated with chronic immune stimulation such as cancer, chronic intracellular infections, chronic pulmonary diseases, autoimmune diseases, and allogeneic transplantation (11).

Older individuals tend to exhibit abundance of late-stage differentiated memory T cells. CD57 is a receptor expressed on CD8^+^ and CD4^+^ T cells in late stages of differentiation (12). Late-differentiated T cells are characterized by the expression of CD45RO, reduced or almost undetectable expression of costimulatory molecules CD27 and CD28, and chemokine receptor CCR7. The re-expression of CD45RA is also characteristic (13).

Previous reports confirmed that CD57^+^CD8^+^ T cells can be defined as “replicatively senescent cells,” although these cells are not “functionally exhausted” (13). The progressive decrease of T cell function as a consequence of a chronically high antigen load is described as part of the phenomenon of exhaustion (14). Nevertheless, senescent CD57^+^CD8^+^ T cells are able to secrete TNF-α and IFN-γ upon encounter with antigen (15).

CD8+CD28^−^ T cells are end-stage cells that have lost the expression of CD28. Lin and colleagues showed that they had the lowest telomerase activity among memory and highly differentiated CD8^+^ T cell subsets. Consistent with this finding, those researchers also demonstrated that CD8^+^CD28^−^ T cells had “the shortest mean telomere length” among the studied groups of memory T cells (16).

Prior studies suggest that the replicative capacity of CD8^+^CD28^−^ T cells in response to antigen stimulation is significantly reduced and the telomere length is shorter in comparison with CD8^+^CD28^+^ T cells (17). High percentages of CD8^+^CD28^−^ T cells are related to reduced response to vaccination (18) and have been associated with mortality in a cohort of elderly Swedish (19).

A study in healthy Cubans showed an effect of gender in the dynamics of T cell subpopulations during aging. Although the proportion of late-differentiated CD8^+^CD28^−^ and CD8^+^CD57^+^ T cells increased with age, it was only statistically significant in males. Cuban women preserve the proportion of CD8^+^CD28^−^ and CD8^+^CD57^+^ T cells practically constant at all ages. Our group also reported other changes with age within the CD8^+^ T cell subset. In this occasion, an increased frequency of terminally differentiated CD8^+^CD45RA^+^CD28^−^ T cells was found in females as they aged, while males showed higher frequency of these cells from youth. By contrast, within CD4^+^ T lymphocytes, terminally differentiated CD4^+^CD45RA^+^CD28^−^ T cells showed an age-associated increase in both sexes, though higher proportions were found in males (4).

The detection of differences concerning the structure and function of the immune system in males and females has been described (20, 21). In addition to hormones, genetic factors can determine the differences in the immune response between males and females. The fact that some genes in the X chromosome are involved in immunity has been addressed (21). It has also been proposed that estrogens improve humoral immunity, whereas androgens and progesterone have a tendency to hamper it (22).

Gender differences in the immune system have been evidenced also in epidemiological studies showing higher incidence of autoimmune diseases in females and higher rate of chronic inflammatory illness such as atherosclerosis-related diseases in males. Nevertheless, the interaction among hormones, genetics, inflammation, and immune system presents a complex scenario that must be more intensively studied (4).

The influence of gender in immunosenescence also appeared in the Berlin Aging Study II, which reported gender-related differences concerning the consequences of age and CMV infection on CD4 and CD8 T cells. This study reported that older men showed higher frequencies of late-differentiated CD8^+^CD57^+^ T cells and concluded that in elderly men, the “CMV-associated senescence of T cells” was more pronounced than in elderly women (23).

The Berlin study also showed a strong effect of CMV infection in the appearance of CD45RA^+^CCR7^−^CD27^−^CD28^−^ terminally differentiated T cells (so-called TEMRA). They observed a significantly lower proportion of CD4^+^CD45RA^+^CCR7^−^CD27^−^CD28^−^ T cells in CMV-negative individuals than in CMV-positive individuals, regardless of gender and age. Concerning the frequency of CD8^+^ T cells, age was observed to have a significant influence, but here too, it was only significant in subjects with demonstrated CMV infection. Therefore, frequency of terminally differentiated T cells was significantly higher in CMV-positive elderly individuals than it was in CMV-negative elderly, notwithstanding gender (23).

Cytomegalovirus is a common herpes virus affecting the 60–90% of the global population. The prevalence of infected individuals increases with age. It is expected that 90% of individuals could be infected nearby the 90 decade of life in contrast with the evidence of 40–60% of individuals in the middle age population (24). The OCTO study reported that the prevalence of subjects with CMV–IgG antibodies in individuals older than 80 years was around 90%, while in middle-aged individuals, it was relatively consistent at 67% (6). Interestingly, our group reported a high seroprevalence of CMV seropositivity greater than 80% in Cuban healthy population from young ages (4).

Persistent infection with viruses such us CMV can augment the accumulation of senescent CD4^+^ and CD8^+^ T cells, identified as CD27^−^CD28^−^CD45RA^+^KLRG1^+^ and CD57^+^, compared to age-matched seronegative individuals (25). The research in this area has been predominantly focused on CD8^+^ T cells, which display decreased naive populations and increased memory subset distribution consistent with a more memory/late effector cell profile. This is accompanied by changes in function such as reduced proliferative capacity, especially in CD57^+^ T cells, and increased cytotoxic and secretory functions (26, 27).

However, changes in T cell subpopulations are also evident in CD4^+^ T lymphocytes. A recent work described the significant increment in the percentages of CD4^+^CD57^+^ T cells in young CMV-positive individuals, compared with young CMV-negative individuals. They showed that CD4^+^ T cells that coexpress CD57 and CD154 are only present in CMV-positive subjects and are considered a very polyfunctional CD4^+^ subset (28). This group had previously revealed an increase in CD8^+^CD57^+^ T cells in CMV-positive young subjects (29).

The CMV-IgG seropositivity was determined in healthy Cubans of all ages in a study conducted by Garcia and colleagues. The general seroprevalence of CMV seropositivity was 90%. More than 90% of elderly individuals had antibodies against CMV, notwithstanding their gender. Nevertheless, young males (93.3%) had higher seroprevalence than young females (73.6%) (4).

In our analysis, the higher percentage of CD45RA^+^CD28^−^ T cells within the CD4^+^ and CD8^+^ subsets described in Cuban males, but not in females, during young ages can be explained by the differential effect of gender and age in the thymic output (4). Furthermore, since androgens and testosterone have higher association with severe thymus involution than female hormones, young males could have less protected immune system than females. This combined with exposure to high antigenic loads since early ages could have driven immunosenescence in young males (4, 30).

The thymic involution in elderly people induces a reduction of naive T cells in the periphery, regardless of CMV infection. Nonetheless, during persistent CMV infection, the memory T cells frequency is higher, possibly because of the latency of CMV, which exerts a persistent stimulation on the immune system in order to control the virus (24, 26). Additionally, CMV reactivation may occur more often in older people (31). From this point, an impairment of the immune system would hamper its capacity to control the CMV infection; therefore, a reactivation of the CMV would lead to a long-lasting antigen stimulation and accelerate the accumulation of CD28^−^ T cells as well as the emergence of the phenomenon of immunosenescence, functioning as a closed loop (24).

## Treatment with Platinum-Based Chemotherapy Entails Different Patterns of Terminally Differentiated CD8 T Cells in NSCLC Patients

Around 70% of cancer-related deaths and 60% of new cancer diagnosis occur in patients older than 65 years (1). Moreover, as aging is a global phenomenon, a rapid increase in elderly with malignancies is expected (32).

A study in cancer patients (respiratory, digestive, reproductive, head, and neck) showed an expansion of CD8^+^CD28^−^ T cells in heavy chemo-treated patients compared with healthy volunteers and treatment-naive patients (33). Another research in patients with various forms of lung cancer receiving chemotherapy reported higher proportions of CD28^−^CD57^+^ cells, thereby highlighting the most pronounced changes in lung cancer patients with stage IV of the disease (34).

Recently, high proportions of CD4^+^CD28^−^ and CD4^+^CD57^+^ T cells have been reported in CMV-positive glioblastoma patients. Additionally, these researchers described short survival in glioblastoma patients with high proportions of CD4^+^CD28^−^ and CD4^+^CD57^+^CD28 null T cells, thereby suggesting an association between those immunosenescence markers and survival in CMV-positive glioblastoma patients (35).

Our group evaluated the presence of the CD28 receptor on CD4^+^ and CD8^+^ T cells in a cohort of Cuban advanced NSCLC patients, before and after administration of first-line platinum-based chemotherapy. We found that the proportion of CD4^+^CD28^−^ T cells significantly increased in NSCLC patients after treatment with platinum-based chemotherapy, compared with healthy volunteers and with cancer patients without chemotherapy. Healthy volunteers and cancer patients without chemotherapy had low proportions of CD8^+^CD28^−^ T cells. Cancer patients treated with standard front-line chemotherapy showed the highest proportions of CD8^+^CD28^−^ T cells (36).

In addition, our group investigated the frequency of CD8^+^CD57^+^ T cells and CD45RA^+^CD28^−^ on CD4^+^ and CD8^+^ T cells in Cuban NSCLC patients after front-line platinum-based chemotherapy. We showed that the frequency of CD8^+^CD57^+^, CD4^+^CD45^+^CD28^−^, and CD8^+^CD45^+^CD28^−^ T cells remained unchanged, regardless of the presence of cancer itself or the chemotherapy treatment.

Based on the high prevalence of CMV infection in Cubans and on previous findings in healthy elderly (4), we hypothesized that notwithstanding cancer disease or chemotherapy, aging and possibly chronic CMV infection could be the main causes for the increase of CD45RA^+^CD28^−^ T cells (36). In our opinion, platinum-based chemotherapy probably causes only the increase of CD28^−^ T cell subpopulations within CD4^+^ and CD8^+^ subsets. Otherwise, changes in the frequency of CD4^+^ or CD8^+^CD45RA^+^CD28^−^ and CD57^+^CD8^+^ T cells seems not to be related with cancer disease or chemotherapy.

Although consensus about necessary chronic antigen stimulation, especially CMV, to cause immunosenescence is under constant discussion, maintaining CMV reactivations under control requires a huge effort from the immune system. This virus could be responsible for the functional impairment of many cell types from innate and adaptive immune systems. Besides affecting the immunosurveillance, this virus could also contribute to the pathogenesis of some inflammatory diseases and even cancer (24).

## CD8^+^CD28^−^ T Cells and CD4/CD8 Ratio as Predictive Biomarkers of Efficacy of Therapeutic Vaccination with the Epidermal Growth Factor (EGF)-Based Vaccine CIMAvax-EGF

The suppression induced by tumor disease and by standard therapies such as chemotherapy and radiation can influence detrimentally the immune system of cancer patients. Nowadays, the assessment of a patient’s immune status represents a valuable tool for determine patients for undergoing immunotherapy (37, 38).

Biomarkers are becoming more necessary in order to select patients who could benefit from therapies, either in the initial phase of the disease or in advanced cancer stages. In such cases, the definition of personalized treatments in tumor disease could lead to an improvement of therapeutic success (39).

CIMAvax-EGF is a therapeutic cancer vaccine developed to generate specific humoral response against the EGF (40). More than 4,000 advanced NSCLC patients have been treated with the CIMAvax-EGF vaccine, which is safe and immunogenic and have proved its efficacy (41).

As previous results published by our Institute showed, a relation between the magnitude of specific anti-EGF antibody response and the clinical outcomes of vaccinated patients has been demonstrated when using CIMAvax-EGF as switch maintenance therapy after platinum-based first-line chemotherapy in NSCLC patients. Young patients showed the best clinical results (42, 43). These results suggest that the clinical benefit in CIMAvax-EGF vaccinated NSCLC patients goes together with the development of a good specific humoral response (36).

In a recent article, our group proposed the frequencies of CD8^+^CD28^−^ T cells and the CD4/CD8 ratio as possible predictive biomarkers for the CIMAvax-EGF efficacy. Consequently, NSCLC patients with a proportion of CD8^+^CD28^−^ T cells of less than 24% and a CD4/CD8 ratio >2 determined after front-line standard chemotherapy and prior to vaccination with CIMAvax-EGF achieved a median survival superior by almost 20 months to that of vaccinated patients with more than 24% of CD8^+^CD28^−^ T cells and a CD4/CD8 ratio <2. These findings emphasize the impact of the immune status on the clinical evolution of CIMAvax-EGF vaccinated NSCLC patients and validate the usefulness of late-stage differentiated CD8^+^ T cells as predictive biomarkers for the CIMAvax-EGF efficacy (36).

As studies of the senescence of the immune system advance, it can be predicted that markers of the dynamics of lymphocytes and cytokines along individual’s lifetime will be increasingly used as prognostic factors and treatment predictors in several diseases. In contrast to genetic markers, which are mainly endogenous individual traits, markers of immunosenescence evolve under extrinsic environmental influences, which obviously vary among diverse human groups. Thus, findings are difficult to extrapolate from country to country. For this reason, specific studies in diverse populations are needed to better asses the timelines of immunosenescence and the influence of chronic antigenic loads and gender. It has been predicted that different forms of immunotherapy will become part of the main therapeutic strategy in an increasing fraction of cancer patients in the near future. This scenario will make specific studies of immunosenescence mandatory.

## Author Contributions

DS, AL, and BG have overall responsibility for writing the paper.

## Conflict of Interest Statement

The authors declare that the research was conducted in the absence of any commercial or financial relationships that could be construed as a potential conflict of interest.

## References

[1] Fulop T Larbi A Kotb R de Angelis F Pawelec G Aging, immunity, and cancer. Discov Med (2011) 11(61):537–50. 21712020

[2] Fulop T Larbi A Kotb R Pawelec G. Immunology of aging and cancer development. Interdiscip Top Gerontol (2013) 38:38–48. 10.1159/000343599 23503514

[3] Kern F Khatamzas E Surel I Frommel C Reinke P Waldrop SL Distribution of human CMV-specific memory T cells among the CD8pos. subsets defined by CD57, CD27, and CD45 isoforms. Eur J Immunol (1999) 29(9):2908–15. 10.1002/(SICI)1521-4141(199909)29:09<2908::AID-IMMU2908>3.0.CO;2-8 10508265

[4] Garcia Verdecia B Saavedra Hernandez D Lorenzo-Luaces P de Jesus Badia Alvarez T Leonard Rupale I Mazorra Herrera Z Immunosenescence and gender: a study in healthy Cubans. Immun Ageing (2013) 10(1):16. 10.1186/1742-4933-10-16 23627933 PMC3667016

[5] Chidrawar S Khan N Wei W McLarnon A Smith N Nayak L Cytomegalovirus-seropositivity has a profound influence on the magnitude of major lymphoid subsets within healthy individuals. Clin Exp Immunol (2009) 155(3):423–32. 10.1111/j.1365-2249.2008.03785.x 19220832 PMC2669518

[6] Olsson J Wikby A Johansson B Lofgren S Nilsson BO Ferguson FG. Age-related change in peripheral blood T-lymphocyte subpopulations and cytomegalovirus infection in the very old: the Swedish longitudinal OCTO immune study. Mech Ageing Dev (2000) 121(1–3):187–201. 10.1016/S0047-6374(00)00210-4 11164473

[7] Pawelec G Hallmarks of human “immunosenescence”: adaptation or dysregulation? Immun Ageing (2012) 9(1):15 10.1186/1742-4933-9-15 22830639 PMC3416738

[8] Pera A Campos C Lopez N Hassouneh F Alonso C Tarazona R Immunosenescence: implications for response to infection and vaccination in older people. Maturitas (2015) 82(1):50–5. 10.1016/j.maturitas.2015.05.004 26044074

[9] Pawelec G T-cell immunity in the aging human. Haematologica (2013) 99(5):795–7. 10.3324/haematol.2013.094383 PMC4008116 24790056

[10] Kouri V Correa CB Verdasquera D Martinez PA Alvarez A Aleman Y Diagnosis and screening for cytomegalovirus infection in pregnant women in Cuba as prognostic markers of congenital infection in newborns: 2007-2008. Pediatr Infect Dis J (2010) 29(12):1105–10. 10.1097/INF.0b013e3181eb7388 20622711

[11] Strioga M Pasukoniene V Characiejus D. CD8+ CD28- and CD8+ CD57+ T cells and their role in health and disease. Immunology (2011) 134(1):17–32. 10.1111/j.1365-2567.2011.03470.x 21711350 PMC3173691

[12] Brenchley JM Karandikar NJ Betts MR Ambrozak DR Hill BJ Crotty LE Expression of CD57 defines replicative senescence and antigen-induced apoptotic death of CD8+ T cells. Blood (2003) 101(7):2711–20. 10.1182/blood-2002-07-2103 12433688

[13] Kared H Martelli S Ng TP Pender SL Larbi A. CD57 in human natural killer cells and T-lymphocytes. Cancer Immunol Immunother (2016) 65(4):441–52. 10.1007/s00262-016-1803-z 26850637 PMC11029668

[14] Akbar AN Henson SM. Are senescence and exhaustion intertwined or unrelated processes that compromise immunity? Nat Rev Immunol (2011) 11(4):289–95. 10.1038/nri2959 21436838

[15] Le Priol Y Puthier D Lecureuil C Combadiere C Debre P Nguyen C High cytotoxic and specific migratory potencies of senescent CD8+ CD57+ cells in HIV-infected and uninfected individuals. J Immunol (2006) 177(8):5145–54. 10.4049/jimmunol.177.8.5145 17015699

[16] Lin J Epel E Cheon J Kroenke C Sinclair E Bigos M Analyses and comparisons of telomerase activity and telomere length in human T and B cells: insights for epidemiology of telomere maintenance. J Immunol Methods (2010) 352(1–2):71–80. 10.1016/j.jim.2009.09.012 19837074 PMC3280689

[17] Effros RB. Replicative senescence: the final stage of memory T cell differentiation? Curr HIV Res (2003) 1(2):153–65. 10.2174/1570162033485348 15043200

[18] Goronzy JJ Fulbright JW Crowson CS Poland GA O’Fallon WM Weyand CM. Value of immunological markers in predicting responsiveness to influenza vaccination in elderly individuals. J Virol (2001) 75(24):12182–7. 10.1128/JVI.75.24.12182-12187.2001 11711609 PMC116115

[19] Wikby A Johansson B Olsson J Lofgren S Nilsson BO Ferguson F. Expansions of peripheral blood CD8 T-lymphocyte subpopulations and an association with cytomegalovirus seropositivity in the elderly: the Swedish NONA immune study. Exp Gerontol (2002) 37(2–3):445–53. 10.1016/S0531-5565(01)00212-1 11772532

[20] Grossman CJ Regulation of the immune system by sex steroids. Endocr Rev (1984) 5(3):435–55. 10.1210/edrv-5-3-435 6381037

[21] Caruso C Accardi G Virruso C Candore G Sex, gender and immunosenescence: a key to understand the different lifespan between men and women? Immun Ageing (2013) 10(1):20 10.1186/1742-4933-10-20 23680476 PMC3737094

[22] Sakiani S Olsen NJ Kovacs WJ. Gonadal steroids and humoral immunity. Nat Rev Endocrinol (2013) 9(1):56–62. 10.1038/nrendo.2012.206 23183675

[23] Di Benedetto S Derhovanessian E Steinhagen-Thiessen E Goldeck D Muller L Pawelec G. Impact of age, sex and CMV-infection on peripheral T cell phenotypes: results from the Berlin BASE-II Study. Biogerontology (2015) 16(5):631–43. 10.1007/s10522-015-9563-2 25732234

[24] Soderberg-Naucler C Fornara O Rahbar A Cytomegalovirus driven immunosenescence – an immune phenotype with or without clinical impact? Mech Ageing Dev (2016) 158:3–13. 10.1016/j.mad.2016.06.005 27318107

[25] Akbar AN Henson SM Lanna A. Senescence of T lymphocytes: implications for enhancing human immunity. Trends Immunol (2016) 37(12):866–76. 10.1016/j.it.2016.09.002 27720177

[26] Pawelec G. Immunosenenescence: role of cytomegalovirus. Exp Gerontol (2014) 54:1–5. 10.1016/j.exger.2013.11.010 24291068

[27] Terrazzini N Kern F. Cell-mediated immunity to human CMV infection: a brief overview. F1000Prime Rep (2014) 6:28. 10.12703/P6-28 24860650 PMC4018181

[28] Pera A Vasudev A Tan C Kared H Solana R Larbi A. CMV induces expansion of highly polyfunctional CD4+ T cell subset coexpressing CD57 and CD154. J Leukoc Biol (2016). 10.1189/jlb.4A0316-112R 27566833

[29] Pera A Campos C Corona A Sanchez-Correa B Tarazona R Larbi A CMV latent infection improves CD8+ T response to SEB due to expansion of polyfunctional CD57+ cells in young individuals. PLoS One (2014) 9(2):e88538. 10.1371/journal.pone.0088538 24533103 PMC3922920

[30] Hince M Sakkal S Vlahos K Dudakov J Boyd R Chidgey A. The role of sex steroids and gonadectomy in the control of thymic involution. Cell Immunol (2008) 252(1–2):122–38. 10.1016/j.cellimm.2007.10.007 18294626

[31] Stowe RP Kozlova EV Yetman DL Walling DM Goodwin JS Glaser R Chronic herpesvirus reactivation occurs in aging. Exp Gerontol (2007) 42(6):563–70. 10.1016/j.exger.2007.01.005 17337145 PMC1992441

[32] Falci C Gianesin K Sergi G Giunco S De Ronch I Valpione S Immune senescence and cancer in elderly patients: results from an exploratory study. Exp Gerontol (2013) 48(12):1436–42. 10.1016/j.exger.2013.09.011 24120567

[33] Chen IH Lai YL Wu CL Chang YF Chu CC Tsai IF Immune impairment in patients with terminal cancers: influence of cancer treatments and cytomegalovirus infection. Cancer Immunol Immunother (2010) 59(2):323–34. 10.1007/s00262-009-0753-0 19685052 PMC11030572

[34] Onyema OO Decoster L Njemini R Forti LN Bautmans I De Waele M Shifts in subsets of CD8+ T-cells as evidence of immunosenescence in patients with cancers affecting the lungs: an observational case-control study. BMC Cancer (2015) 15:1016. 10.1186/s12885-015-2013-3 26711627 PMC4692066

[35] Fornara O Odeberg J Wolmer Solberg N Tammik C Skarman P Peredo I Poor survival in glioblastoma patients is associated with early signs of immunosenescence in the CD4 T-cell compartment after surgery. Oncoimmunology (2015) 4(9):e1036211. 10.1080/2162402X.2015.1036211 26405601 PMC4570117

[36] Saavedra D Garcia B Lorenzo-Luaces P Gonzalez A Popa X Fuentes KP Biomarkers related to immunosenescence: relationships with therapy and survival in lung cancer patients. Cancer Immunol Immunother (2016) 65(1):37–45. 10.1007/s00262-015-1773-6 26589409 PMC11028799

[37] Gustafson MP Lin Y LaPlant B Liwski CJ Maas ML League SC Immune monitoring using the predictive power of immune profiles. J Immunother Cancer (2013) 1:7. 10.1186/2051-1426-1-7 25512872 PMC4266565

[38] Chang S Kohrt H Maecker HT. Monitoring the immune competence of cancer patients to predict outcome. Cancer Immunol Immunother (2014) 63(7):713–9. 10.1007/s00262-014-1521-3 24487923 PMC4058358

[39] Berghella AM Contasta I Lattanzio R Di Gregorio G Campitelli I Marino S The role of gender-specific cytokine pathways as drug targets and gender-specific biomarkers in personalized cancer therapy. Curr Drug Targets (2016). 27397062 10.2174/1389450117666160630173647

[40] Rodriguez PC Popa X Martinez O Mendoza S Santiesteban E Crespo T A phase III clinical trial of the epidermal growth factor vaccine CIMAvax-EGF as switch maintenance therapy in advanced non-small cell lung cancer patients. Clin Cancer Res (2016) 22(15):3782–90. 10.1158/1078-0432.CCR-15-0855 26927662

[41] Crombet Ramos T Rodriguez PC Neninger Vinageras E Garcia Verdecia B Lage Davila A. CIMAvax EGF (EGF-P64K) vaccine for the treatment of non-small-cell lung cancer. Expert Rev Vaccines (2015) 14(10):1303–11. 10.1586/14760584.2015.1079488 26295963

[42] Garcia B Neninger E de la Torre A Leonard I Martinez R Viada C Effective inhibition of the epidermal growth factor/epidermal growth factor receptor binding by anti-epidermal growth factor antibodies is related to better survival in advanced non-small-cell lung cancer patients treated with the epidermal growth factor cancer vaccine. Clin Cancer Res (2008) 14(3):840–6. 10.1158/1078-0432.CCR-07-1050 18245547

[43] Neninger Vinageras E de la Torre A Osorio Rodriguez M Catala Ferrer M Bravo I Mendoza del Pino M Phase II randomized controlled trial of an epidermal growth factor vaccine in advanced non-small-cell lung cancer. J Clin Oncol (2008) 26(9):1452–8. 10.1200/JCO.2007.11.5980 18349395

